# Production potential of the first generation of selected Pitalah and Bayang ducks as a community economic resource in West Sumatra

**DOI:** 10.5455/javar.2023.j690

**Published:** 2023-09-24

**Authors:** Zasmeli Suhaemi, Sabrina Sabrina, Nita Yessirita, Nelzi Fati, Febriani Febriani, Burhanudin Malik

**Affiliations:** 1 Department of Agribusiness, Faculty of Science, University of Nahdlatul Ulama of West Sumatra, Padang, Indonesia; 2 Faculty of Animal Science, Andalas University, Padang, Indonesia; 3 Faculty of Agriculture, Ekasakti University, Padang, Indonesia; 4 Department of Animal Science, Payakumbuh State Polytechnic of Agriculture, Payakumbuh, Indonesia; 5 Faculty of Economic, Tamansiswa University, Padang, Indonesia; 6 Department of Animal Science, Faculty of Agriculture, Djuanda University, Bogor, Indonesia

**Keywords:** Local duck breed, genetic improvement, lipid profile, selection

## Abstract

**Objectives:**

This study aimed to measure the production potential of selected Pitalah and Bayang male ducks and their first generation.

**Materials and Methods:**

A total of 100 Pitalah and 100 Bayang 1-day-old ducks (40 males, 60 females) were obtained from local farmers and reared for 32–34 weeks. Twenty male ducks were selected as parental ducks based on their body weight (BW) and feed conversion ratio (FCR) at weeks 8 and 12. Forty female layer ducks were selected as parental ducks based on their blood cholesterol levels. Selected parental ducks were allowed to reproduce, and the ducklings were reared for 8 weeks before their BW, BW gain (BWG), feed intake, FCR, carcass weight percentage, abdominal fat percentage, and income over feed and duck cost (IOFC) ratio were measured. The data were subjected to a *t*-test.

**Results:**

Pitalah parental and first-generation ducks had better production performance and blood lipid profiles than Bayang ducks (*p *< 0.05). Based on the IOFC ratio, rearing Pitalah ducks for 8 weeks for meat production was more profitable and beneficial as a community economic resource.

**Conclusion:**

The selection of Pitalah and Bayang ducks was worth pursuing, as the G1 of both Pitalah and Bayang ducks had better production performance in terms of their BW, BWG, and FCR. Based on the IOFC values, raising Pitalah ducks for 8 weeks for meat production would be more economically beneficial.

## Introduction

Indonesia is considered a country with the highest biodiversity in the world. This includes local poultry breeds, which in developing countries, contribute to almost 95% of the total poultry population and play an important role in the poultry industry. Being well adapted to local animal rearing systems and environmental conditions, these local poultry animals, including ducks, deserve to be intensively studied to improve their use and conservation [1–3].

Although ducks are mostly reared for egg production, demand for duck meat also increases. Duck meat is a nutritious food as it contains high protein with a complete amino acid composition, high polyunsaturated fatty acids, and a balanced ratio of omega-6 and omega-3 fatty acids [4]. However, the meat of local ducks tends to have a high cholesterol content [5], which contradicts the increasing health consciousness of consumers. Duck meat producers are required to provide nutritious and healthy meat products.

Efforts to reduce duck meat’s cholesterol content have been made. The use of plant materials containing secondary metabolites with antilipidemic properties has been extensively studied [6,7]. Another way to improve the quality of livestock and its products is through genetic improvement through selection and mating systems [8]. The success of the latter is indicated by an increase in the appearance of production traits [9].

Pitalah and Bayang ducks are West Sumatra local breeds recognized as local duck breeds with an important socio-economic role as livelihood providers for smallholder farmers. These ducks are known for their strong ability to survive harsh environmental conditions with rough and alternative fodder [3]. In addition, they are found to have good egg and meat production and relatively lower blood cholesterol levels [10]. Yet, their potential has not been fully developed. Therefore, this study was conducted to improve the genetic quality of Pitalah and Bayang ducks to be used as economic resources for the community.

## Materials and Methods

### Ethical approval

The animals were raised and treated according to Indonesian Government Regulation (PP) number 95 year 2012 on Veterinary Public Health and Animal Welfare.

### Animals 

A total of 200 1-day-old ducks (DOD) of Pitalah breed (40 males, 60 females) with an average body weight (BW) of 38.47 ± 1.94 gm and Bayang breed (40 males, 60 females) with an average BW of 38.84 ± 1.53 gm were obtained from breeder farmers in Pitalah Village, Batipuh District, Tanah Datar Regency, and Ganting Village, Bayang District, Pesisir Selatan Regency, West Sumatra. The ducks were raised until they were 34 weeks old and laid their eggs. The eggs were left to hatch, and the ducklings were raised for 8 weeks as the first generation (G1).

### Rations 

Ducks were fed rations formulated from a mixture of commercial concentrate, corn meal, and rice bran meal. Rations were made for the starter (0–4 weeks), grower (0–4 weeks), and finisher (>5 weeks) phases. The composition and nutrient contents of feeds and rations are presented in Tables 1 and 2.

### Selection of parental ducks

The selection of male parental (F0) ducks was done when the ducks reached 8 and 12 weeks of age. Twenty male ducks of each breed with the best BW and feed conversion ratio (FCR) were selected (F0) and reared for 32 weeks. For female ducks, selection of F0 ducks was done when the ducks were already in their layer phase (32–34 weeks). Forty female ducks with the lowest blood and egg cholesterol levels were selected.

**Table 1.** Nutrient contents of feeds. Source: [11].

Nutrients	Rice bran	Corn meal	Concentrate
Dry matter (%)	90.70	91.29	89.63
Crude protein (%)	11.19	8.60	31.00
Crude fiber (%)	17.63	3.37	5.00
Crude fat (%)	4.00	2.60	3.00
Metabolizable energy (kcal/kg)	1,630	3,420	2,600

**Table 2.** Composition and nutrient contents of rations.

Feeds/nutrients	Starter and Finisher	Grower
	(%)	(%)
Corn meal	40	45
Rice bran	20	25
Commercial concentrate	40	30
Crude Protein	18.20	16.14
Crude lipid	3.14	3.11
Crude Fiber	8.14	8.02
Metabolizable energy (kcal/kg)	2,629	2,685

Calculated based on the data in Table 1.

### Blood collection

Blood samples were collected from male (aged 8 weeks) and female (aged 32–34 weeks) F0 ducks and G1 ducks (8 weeks) of both breeds. Blood was obtained from the wing vein using sterilized syringes, and the serum was isolated within 4–6 h. Blood serum samples were stored at −5°C for pending cholesterol analysis.

### Lipid profile determination 

Blood lipid profiling of F0 and G1 ducks was conducted by determining total cholesterol, triglycerides, low-density lipoprotein (LDL), and high-density lipoprotein (HDL) contents. Lipid profile determination was conducted using the cholesterol oxidase-peroxidase 4 aminoantipyrine method with a photometric system. For cholesterol and triglyceride contents, distilled water (blank), blood serum, and a standard solution of 10 µl each were prepared. Each was mixed with 1,000 µl reagent, homogenized, and incubated for 20 min at room temperature. The mixture was further read using a Microlab 300 photometer at 546 nm wavelength.

Filtrates for LDL and HDL were prepared by mixing distilled water (blank), blood serum, and a standard of 10 µl each with 1,000 µl reagent. The mixture was homogenized on a vortex mixer before being incubated for 30 min. The mixture was further centrifuged for 15 min at 2,500 rpm, and the filtrate was separated from the precipitate. The filtrate (100 µl) was mixed with distilled water (blank, 100 µl), standard solution (10 µl), and cholesterol reagent (1,000 µl), and a homogenous mixture was obtained. The mixture was incubated for 20 min at room temperature before it was read using a Microlab 300 photometer at 546 nm wavelength.

### Production performance

**Figure 1.**
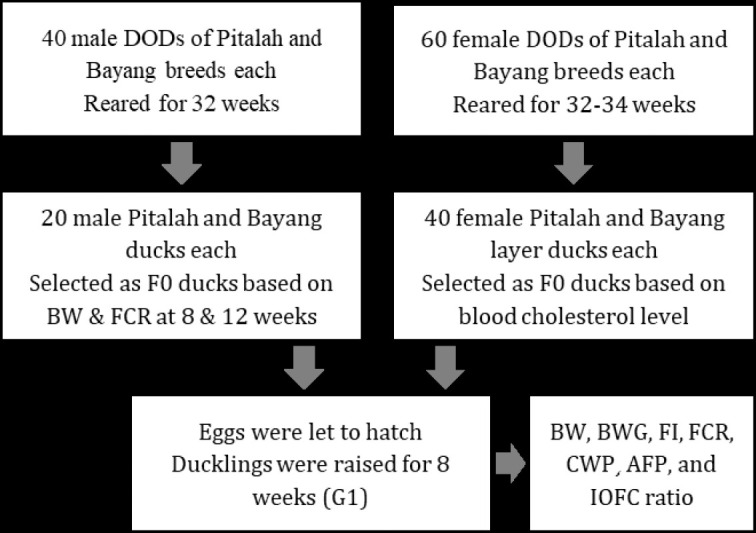
Flow chart of methods of the study.

Production performance was assessed based on BW, BW gain (BWG), feed intake (FI), FCR, carcass weight percentage (CWP), abdominal fat percentage (AFP), and income over feed and duck cost (IOFC) ratio. Measurements of BW and BWG were taken on F0 ducks aged 8 and 12 weeks and G1 ducks aged 8 weeks. BW was the weight of the ducks before they were slaughtered at the end of the rearing period (8 and 12 weeks), and BWG was the difference between BW and DOD weight. The methods of the study are depicted in Figure 1.

### Statistical analysis

Data of all parameters, excluding IOFC, were subjected to a *t*-test using Statistical Package for the Social Sciences (SPSS) 17.0 (SPSS Inc., USA).

## Results and Discussion

### External performance

**Figure 2.**
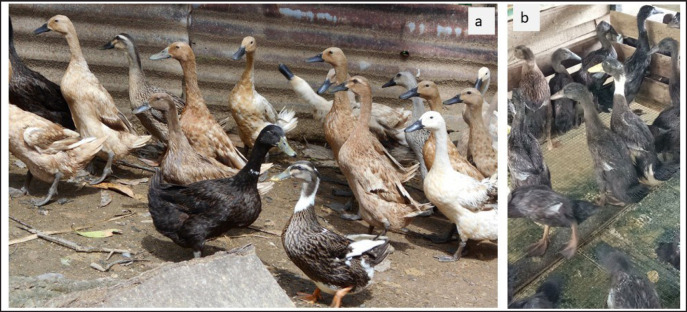
(a) Pitalah ducks with varied external performance in local breeders and (b) Pitalah ducks with more uniform plumage color used in this study.

**Figure 3.**
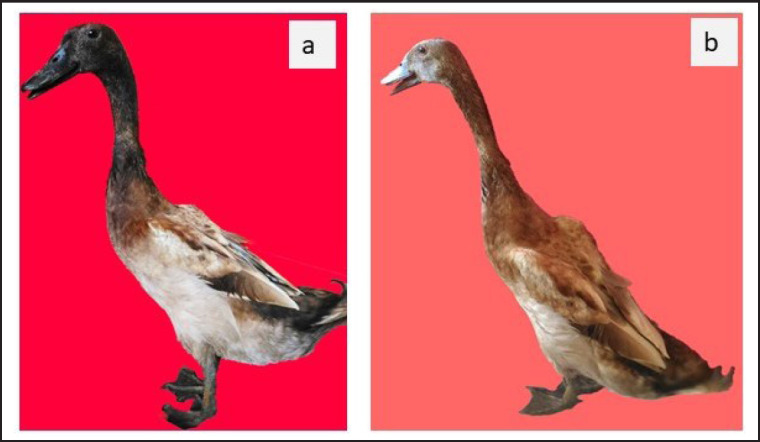
Selected Pitalah ducks: (a) male and (b) female.

**Figure 4.**
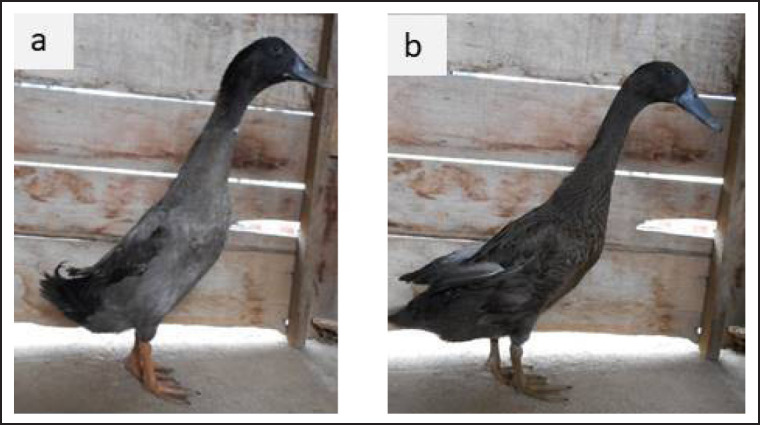
Selected Bayang ducks: (a) male and (b) female.

The external performance of Pitalah and Bayang ducks in local breeders where the ducks used in this originated was remarkably varied (Fig. 2a). The ducks in this study had relatively more uniform plumage colors (Fig. 2b). Selected Pitalah male ducks had dominant gray plumages, a black head, and ivory and yellow feet (Fig. 3a). They selected Pitalah female ducks with gray plumages, a black head, and black legs with ivory and yellow spots on their feet (Fig. 3b). Selected Bayang male ducks had dominant brown plumages, a black head, and black and ivory legs (Fig. 4a). The selected female Bayang ducks had dominant light brown plumages, a dark head, and black legs with ivory spots (Fig. 4b). The findings in this study were somewhat in agreement with what was found by Maharani et al. [12], that highland ducks (Pitalah) tended to have dark plumage colors. In contrast, lowland ducks (Bayang) had relatively light brown plumage. In another study by Rusfidra et al. [13], it was also revealed that Pitalah ducks had darker plumage colors than Bayang ducks.

### Production performance 

Parental (F0) Pitalah and Bayang ducks aged 8 weeks had no different BW, BWG, FI, CWP, or AF. The only significant difference was their FCR, in which Pitalah ducks had better feed efficiency. At 12 weeks, both breeds had significantly different (*p *< 0.05) BW, BWG, FCR, and AFP. No differences were found in FI and CWP (Table 3).

The BW of parental Pitalah ducks (F0) in this study ranged from 1,243.30 (8 weeks) to 1,436 gm (12 weeks), which was much higher than that (887.35 gm) of Pitalah ducks (8 weeks) fed a ration containing 2,600 kcal/kg energy and 18% crude protein [14]. Parental Bayang ducks (F0) had a BW range of 1,204 (8 weeks) to 1,400.7 gm (12 weeks). In other studies, Pitalah and Bayang ducks were found to have BWs of 1,335.06 (8 weeks), 1,466.46 (12 weeks), 1,316.51 (8 weeks), and 1,410.62 gm (12 weeks), respectively [15]. Female Bali ducks (8 weeks) fed rations containing protein containing 2,596 kcal/kg energy and 17.51% crude protein had about 1,136 gm BW [16]. Mojosari-alabio crossbred ducks (8 weeks) had 1,425.72 gm BW [17]. Selected and unselected male Muscovy ducks were raised, and their growth performances were compared [18]. Selected Muscovy ducks were found to have higher BW (4,644 and 5,156 gm) than unselected ones (3,148 and 3,519 gm) at 10 and 12 weeks of age, respectively. In a breeding trial experiment with Peking ducks over seven generations, selected ducks, at the age of 8 weeks, were found to have 18.2% higher BW (3,287 gm) than did the control ducks (2,782 gm) [19]. In this study, G1 Pitalah and Bayang ducks had 6.35% and 8.28% higher BW than their parental ducks, respectively.

**Table 3.** Production performance of F0 and G1 ducks.

Parameter	F0 (8 weeks)		F0 (12 weeks)		G1 (8 weeks)	
	Pitalah	Bayang	Pitalah	Bayang	Pitalah	Bayang
BW (gm)	1,243.30 ± 85.17	1,204.86 ± 06.42	1,436.27 ± 60.93^a^	1,400.71 ± 104.04^b^	1,322.25 ± 37.91	1,304.63 ± 92.04
BWG (gm)	1,204.84 ± 85.07	1,166.03 ± 104.97	1,397.81 ± 65.70^a^	1,361.87 ± 102.61^b^	1,280.87 ± 53.70	1,263.23 ± 87.61
FI (gm)	5,183.50 ± 30.28	5,221.50 ± 52.42	9,099.74 ± 0.30	9,138.15 ± 67.40	5,304.70 ± 54.28	5,430.99 ± 76.40
FCR	4.30 ± 0.28^a^	4.48 ± 0.42^b^	6.51 ± 0.30^a^	6.71 ± 0.52^b^	4.13 ± 0.28^a^	4.30 ± 0.46^b^
CWP (%)	62.64 ± 2.32	62.79 ± 1.78	64.91 ± 3.03	64.09 ± 2.24	63.34 ± 0.50	63.03 ± 1.29
AFP (%)	0.46 ± 0.37	0.30 ± 0.28	1.00 ± 0.28^a^	0.64 ± 0.39^b^	0.37 ± 0.36^a^	0.43 ± 0.23^b^

Means within a row of the same duck age with different superscript are significantly different (*p *< 0.05), F0: parental, G1: first generation, BW: body weight, BWG: body weight gain, FI: feed intake, FCR: feed conversion ratio, CWP: carcass weight percentage, AFP: abdominal fat percentage.

The FCR values of both Pitalah (6.51) and Bayang (6.71) F0 ducks in this study reinforced the findings of other studies that ducks older than 8 weeks had lower feed efficiency. Female Bali ducks aged 14 weeks had an FCR of 6.17 [16], and Korean native ducks aged 6–8 weeks had an FCR of 9.37 [20]. In this study, the FCR of G1 ducks (8 weeks) was both in Pitalah (4.30–4.13) and Bayang (4.48–4.30) populations. This indicated that the selection conducted in this study was in the right direction. In a selection for FCR involving 2,189 Pekin ducks within 11 generations, it was found that FCR (4–7 weeks) improved from 3.305 to 2.847 (0.458 points) with a heritability rate of 5.2 [19]. This improved FCR was caused by reduced carcass fat content, which was in line with the higher CWP and lowered abdominal fat content of G1 ducks in this study.

**Table 4.** Income over feed and DOD (IOFD) cost.

Variable	8 weeks		12 weeks	
	Pitalah	Bayang	Pitalah	Bayang
Ration cost (Rp)	28,018.41	28,230.45	48,986.49	49,198.53
DOD cost (Rp)	5,000.00	5,000.00	5,000.00	5,000.00
Duck selling price (Rp)	49,732.10	48,194.48	57,450.96	56,028.32
IOFC cost (Rp)	16,713.69	14,964.03	3,464.47	1,829.79

Calculation was conducted based on the prevailing prices at the time of the study.

**Table 5.** Lipid profile of F0 and G1 duck blood serum (mg/dl).

Variable	F0		G1	
	Pitalah	Bayang	Pitalah	Bayang
Cholesterol	162.70 ± 3.73^a^	177.54 ± 8.88^b^	162.40 ± 2.50^a^	177.27 ± 6.97^b^
Triglycerides	111.54 ± 6.73^a^	106.82 ± 5.47^b^	111.05 ± 3.56^a^	106.12 ± 3.07^b^
LDL	57.32 ± 5.49^a^	65.44 ± 10.19^b^	41.08 ± 2.40^a^	44.81 ± 2.47^b^
HDL	78.29 ± 6.69	85.50 ± 12.49	84.73 ± 4.65	83.38 ± 5.93

Means within a row with different superscripts are significantly different (*p *< 0.05), F0: parental duck, G1: first generation duck.

**Figure 5.**
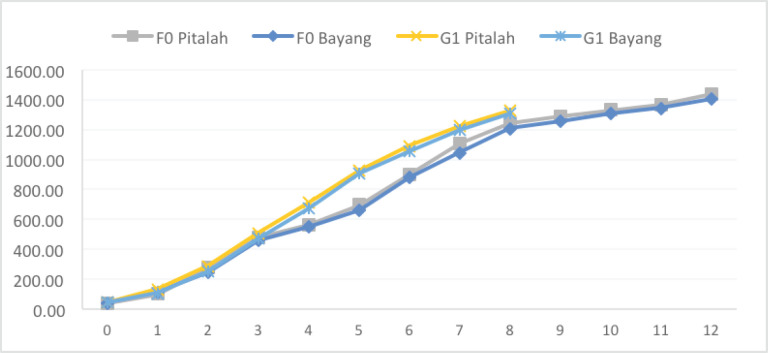
BW of parental (F0) and first generation (G1) Pitalah and Bayang ducks.

The cost of rations for the breeding of Pitalah ducks was higher than that of Bayang ducks (Table 4). This was attributed to the higher FI the Bayang ducks had (Table 3). Production costs were calculated based on the prices of feed, DOD, and products prevailing when the study was conducted. The prices were Rp5,580 per kg of feed for the starter phase (1–4 weeks), Rp5.349 per kg of feed for the grower phase (5–12 weeks), Rp5,000 for DOD, and Rp40,000 per kg of BW. It was revealed in this study that raising Pitalah and Bayang ducks for up to 8 weeks resulted in the highest profit (Rp16,713.69 per head). This result was in line with the findings of other studies, which showed that the best growth rate of ducks of various breeds occurs at 8–10 weeks [16,21].

The best production performance of F0 ducks was obtained at 8 weeks (Tables 3 and 4); the same measurements were taken with G1 ducks of the same age. The BW (1,322.25 and 1,304.63 gm) and BWG (1,280.87 and 1,263.23 gm) of G1 ducks of Pitalah and Bayang breeds were found to be higher than the BW (1,243.30 and 1,204.86 gm) and BWG (1,204.84 and 1,166.03 gm) of their Pitalah and Bayang F0 ducks, respectively. The faster growth rate of G1 ducks of both breeds (Figure 5) in this study was affirmed by the findings that parental selection has succeeded in improving the growth of G1 and G2 of Alabio [22] and Pegagan [8] ducks.

### Blood lipid profiles

Total blood cholesterol, triglycerides, LDL, and HDL contents of F0 and G1 ducks are listed in Table 5. At the parental level, compared to Bayang duck, Pitalah duck had lower serum cholesterol (177.54 *vs.* 111.54 mg/dl), LDL (65.44 *vs.* 57.32 mg/dl), and higher triglycerides (106.82 *vs.* 111.54 mg/dl). No difference was found in serum HDL (85.50 *vs.* 78.29 mg/dl) contents in Bayang and Pitalah ducks, respectively. A similar trend was found in first-generation ducks, in which serum cholesterol (162.40 *vs. *177.27 mg/dl) and LDL (41.08 *vs.* 44.81 mg/dl) contents were found to be lower in Pitalah than in Bayang ducks. In contrast, serum triglyceride content in Pitalah ducks (111.05 mg/dl) was higher than that (106.12 mg/dl) in Bayang ducks. Serum HDL contents (85.50 *vs.* 78.29 mg/dl) in Bayang and Pitalah ducks were not different, respectively.

Blood serum lipid profiles obtained in this study were much lower than those obtained in a study [23], which found that crossbred Peking and Khaki Campbell ducks fed basal ration had cholesterol, triglycerides, LDL, and HDL contents of 203.33, 155.33, 60.87, and 111.40 mg/dl, respectively. In Tegal hen ducks, the figures were found to be 253.70, 235.71, 188.44, and 65.27 mg/dl for blood cholesterol, triglycerides, LDL, and HDL contents, respectively [24]. However, compared to those of P0 ducks, no improvement in blood lipid profiles in G1 ducks was observed in this study.

## Conclusion

The selection of Pitalah and Bayang ducks was worth pursuing, as the G1 of both Pitalah and Bayang ducks had better production performance regarding their BW, BWG, and FCR. Based on the IOFC values, raising Pitalah ducks for 8 weeks for meat production would be more economically beneficial. The results of this study have provided the basis for future selection programs for local ducks, particularly Pitalah and Bayang ducks, as a potential economic resource for the community.

## List of abbreviations

AFP, abdominal fat percentage; BW, body weight; BWG, body weight gain; CWP, carcass weight percentage; dl, deciliter; DOD, one-day-old duck; F0, parental; FCR, feed conversion ratio; G1, first-generation; gm, gram; HDL, high-density lipoprotein; IOFC, income over feed and duck cost; kcal, kilocalorie; kg, kilogram; LDL, low-density lipoprotein; mg, milligram; and µl, microliter.
